# Cellular Senescence and Ageing: Mechanisms and Interventions

**DOI:** 10.3389/fragi.2022.866718

**Published:** 2022-03-29

**Authors:** Andreas Mylonas, Ana O’Loghlen

**Affiliations:** Epigenetics & Cellular Senescence Group, Blizard Institute, Barts and the London School of Medicine and Dentistry, Queen Mary University of London, London, United Kingdom

**Keywords:** senescence, ageing, SASP, age-related disease, hallmarks, senolytics, senomorphics, extracellular vesicles

## Abstract

The influence of the activation of a cellular phenotype termed senescence and it’s importance in ageing and age-related diseases is becoming more and more evident. In fact, there is a huge effort to tackle these diseases via therapeutic drugs targeting senescent cells named senolytics. However, a clearer understanding of how senescence is activated and the influence it has on specific cellular types and tissues is needed. Here, we describe general triggers and characteristics of senescence. In addition, we describe the influence of senescent cells in ageing and different age-related diseases.

## Introduction

Multicellular organisms have been living on our planet for over 750 million years. Multicellularity is a very efficient way of living, as tasks for survival are divided into different cell types. A plant, for example, has different specialized cell types, each regulating a special function, such as taking nutrients from the soil, photosynthesising, or creating a hard trunk for protection. This successful coordination is accomplished due to a simple principle, i.e., the survival and well-being of the multicellular organism are more important than the survival of any individual cell (Michod, 2007; Pichugin et al., 2019).

When a single cell malfunctions, it can potentially harm the whole multicellular organism. There are two ways of controlling such incidences. The first is based on the ability of specialised cells to recognise the malfunctioning cell and destroy it. The second way is based on the ability of the cell to recognize its own dysfunction and cause its own death and/or limit its ability to grow. Mechanisms combining both ways of elimination are used in most cases (Michod, 2007; Pichugin et al., 2019). The induction of cellular senescence is an example of such mechanisms occurring in mammals. First, by recognizing its own dysfunction and inducing a stable cell cycle arrest via activation of cell cycle inhibitors such as p16^INK4A^ and p21^CIP1^. Second, by discharging signals to the immune system so that it recognises and destroys the damaged - senescent – cell (Lee and Schmitt, 2019).

The presence of senescent cells has both beneficial and detrimental effects on the organism. On the one hand, senescence has beneficial outcomes, especially during the early stages of development. Senescence occurs in many different locations during the development of the mammalian embryo and is responsible for the appropriate tissue remodeling and the elimination of unwanted cells as the tissues develop (Munoz-Espin and Serrano, 2014). Senescence is also responsible for beneficial outcomes on the adult organism. For example, it regulates proper wound healing by limiting the development of fibrotic tissue. Senescence induction in myofibroblasts prevents the upregulation of matrix degrading components and creates an anti-fibrotic microenvironment, contributing to normal tissue healing (Jun and Lau, 2010). This mechanism is also conserved during fibrosis in many tissues (Munoz-Espin and Serrano, 2014). On the other hand, senescence is thought to be one of the reasons cell tissues age and overaccumulation of senescent cells usually promotes accelerated biological ageing and age-related diseases, leading to the overall ageing of the organism (van Deursen, 2014). It is hypothesized this is due to either an aged immune system, which inefficiently eliminates senescent cells from tissues, or due to an incompetent SASP released by senescent cells. It is probable that a combination of both, together with other unknown factors, are implicated in the detrimental effects of the accumulation of senescent cells.

## Triggers of Senescence

**FIGURE 1:**
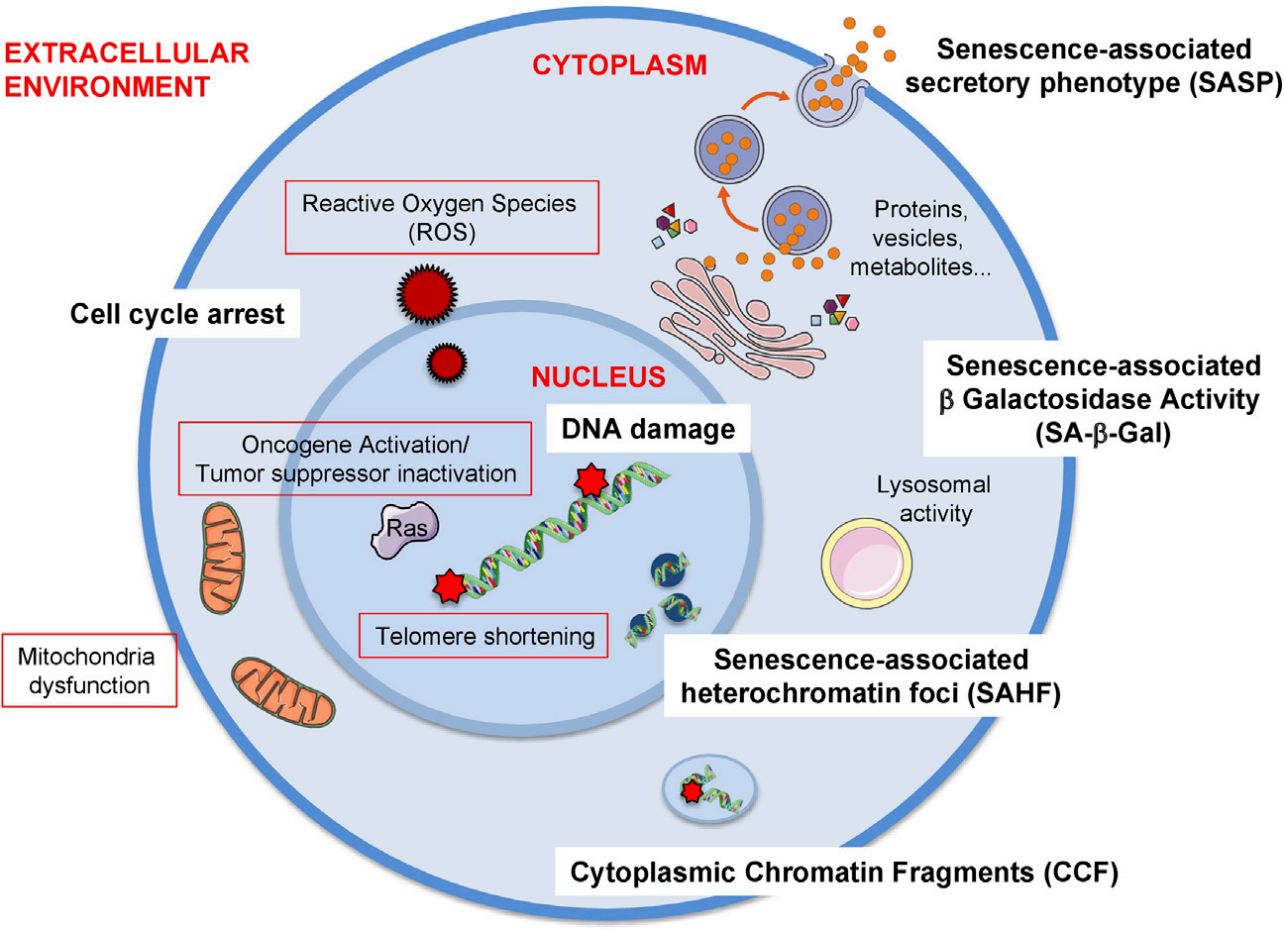
**Triggers and biomarkers of cellular senescence**. There are several stimuli or triggers that activate cellular senescence (red outline). Some of these are depicted in the figure such as the formation of Reactive Oxygen Species (ROS) both from external factors or internal such as mitochondrial dysfunction. Others include the expression of certain oncogenes, e.g., RAS (Rat sarcoma virus) or the loss of tumour suppressor genes, e.g., PTEN (Phosphatase And Tensin Homolog). The shortening of telomeres due to the lack of telomerase enzyme also elicits cellular senescence. Additionally, mitochondrial dysfunction, which can be due to mitochondrial malfunction, increase in mitochondrial size or mass, mitochondrial fusion or mitochondrial fragmentation can also induce senescence. As there is no gold standard biomarker of senescence, a combination of several biomarkers are used to identify this cellular phenotype both *in vitro* and *in vivo*. Some of these biomarkers are the release of a senescence-specific secretome, the senescence-associated phenotype (SASP) formed by proteins, vesicles, metabolites. Other biomarkers the presence of DNA damage and the establishment of a stable cell cycle arrest. Furthermore, chromatin alterations such as heterochromatin foci (senescence-associated heterochromatin foci, SAHF) or the presence of chromatin in the cytoplasm (cytoplasmic chromatin fragments, CCF) are also present during senescence. Finally, the most extensively used biomarker of senescence is the presence of senescence-associated -β- galactosidase activity (SA-β-Gal) which is due to an increase in lysosomal activity, although it is important to take into account that this feature is not exclusive of senescent cells.

Senescence can be triggered by a variety of stresses including but not limited to telomere shortening, oncogene activation and the presence of reactive oxygen species (Figure 1).

### Telomere Shortening

Telomeres are heterochromatic repeated sequences of nucleotides at both ends of human chromosomes, consisting of 8–12 kilobases at birth. With each DNA replication, 50–200 base pairs of telomeres are lost from each human cell, due to the inability of DNA polymerase to replicate the whole molecule. Telomeres shorten with each cell division until they reach a critical point. As a result, a DNA damage response (DDR) is elicited, which in turn increases p16^INK4A^ and p21^CIP1^ cellular levels finally promoting senescence (Di Micco et al., 2021). This type of senescence is called replicative senescence because it originates from the number of replications a cell line undergoes (Deng et al., 2008; Fafian-Labora and O'Loghlen, 2020).

### Oncogene Activation

Oncogene overexpression and tumour suppressor gene inactivation promote oncogene-induced senescence (OIS). Oncogenes are mutated forms of normal genes present in the human genome, called proto-oncogenes. Under normal circumstances, these genes regulate physiological functions favourable to the cells, but when mutated by gene overexpression or amplification they have the potential to promote cancer development. Tumour suppressor genes code for proteins regulating pathways that contribute to the prevention of cancer development. Thus, their loss of function leads to the loss of these cancer-protective properties, causing cancer. Oncogenes known to be overexpressed in OIS include RAS, BRAF, AKT, E2F1 and cyclin-E. Tumour suppressor genes commonly lost in OIS are PTEN and NF-1 (Gil and Peters, 2006; Perez-Mancera et al., 2014; Lee and Schmitt, 2019).

### Mitochondrial Dysfunction

Reactive oxygen species (ROS) is a group of molecules, including hydrogen peroxide (H_2_O_2_), superoxide ion (O_2_^•-^) and hydroxyl radical (^•^OH). They are products of oxidative metabolism in mitochondria, usually scavenged by the enzyme superoxide dismutase (SOD). When mitochondria malfunction, ROS are released causing oxidative damage to mitochondrial and cellular DNA (Desdin-Mico et al., 2020; Di Micco et al., 2021; Martini and Passos, 2022). ROS can also form from the interaction of exogenous factors, such as UV radiation and chemicals from tobacco, and damage cellular DNA. These reactions signal a DDR similar to that caused by telomere shortening, activating p21^CIP1^ and p16^INK4A^ and causing senescence (Di Micco et al., 2021; Prasanna et al., 2021).

## Hallmarks of Senescence

Senescence is a cellular state that can be induced by different stimuli and it is characterised by a stable cell cycle arrest, a secretory-associated phenotype (SASP), macromolecular damage, deregulated metabolism and characteristically-altered cellular morphology. However, as no single marker of senescence is reliable, a combination of markers must be used to identify the senescence phenotype (Sharpless and Sherr, 2015; Gorgoulis et al., 2019) (Figure 1).

### Cell Cycle Arrest

Triggers of senescence activate specific alterations in intracellular pathways to achieve a stable cell cycle arrest. It can be initiated from the *INK4A-ARF* locus, present on chromosomal region 9p21, containing the tumour suppressor genes *INK4A* and *ARF*. Gene expression of the locus is normally silenced by Polycomb Repressive Complexes 1 and 2 (PRC1 and 2). PRC1/2 disruption causes gene activation and transcription of two proteins: p16^INK4A^ and p14^ARF^. Such disruptions are associated with cancer development and trigger oncogene-induced senescence to prevent it (Gil and O'Loghlen, 2014). p16^INK4A^ inhibits the action of the Cyclin-Dependent kinase (CDK4/6) complex, which also hampers phosphorylation of retinoblastoma (Rb) establishing cell cycle arrest (Herranz and Gil, 2018). Finally, p14^ARF^ is also encoded from the *INK4A-ARF* locus and acts by inhibiting Mouse Double Minute-2 homolog protein (MDM2) that activates p53, furtherly promoting cell cycle arrest (Herranz and Gil, 2018).

Furthermore, after DNA damage, two serine/threonine kinases are activated, named Ataxia-Telangiectasia Mutated (ATM) and ATM- and RAD3-related Protein (ATR). These kinases activate Checkpoint Kinases-1 and -2 (CHK-1 and -2), which in turn phosphorylate p53. This is a transcription factor normally bound to MDM2, a ubiquitin-ligase enzyme that tags p53 for degradation by proteasomes. Phosphorylation of p53 causes its release from MDM2, creating a stable and active form of p53, free to travel in the nucleus and upregulate *CDKN1A* transcription and, thus, p21^CIP1^ induction. p21^CIP1^ protein then acts as a CDK inhibitor, blocking G1/S phase cell cycle CDKs, a complex of Cyclin E and CDK2, and S phase CDKs, a complex of Cyclin A and CDK2 or CDK1. CDK2 is a kinase responsible for the phosphorylation of Rb and subsequent inactivation of this protein. This enables the E2 transcription factor (E2F) to regulate the genes needed to promote cell cycle progression. Inhibition of CDK2 hampers the phosphorylation of Rb which traps E2F avoiding the interaction with its target genes and finally establishing cell cycle arrest (Herranz and Gil, 2018).

### Macromolecular Modifications

Senescence is characterised by specific forms of macromolecular damage, for example telomere-associated foci (TAF). DNA damage in TAF is linked to senescence and is expressed by increased phosphorylation of histone-2, in the form of γ-H2AX within telomeres (Martini and Passos, 2022). During senescence, senescence-associated heterochromatin foci (SAHF) can also be formed, characterised by dense chromosomes, concentrated in a single nucleolus and bound to hypoacetylated histones and heterochromatin proteins, like HP1. This chromosomal arrangement in this focus causes the silencing of the expression of the corresponding genes, in particular E2F target genes involved in cell cycle progression (Narita et al., 2003).

Cytoplasmic chromatin fragments (CCFs) are fragments of chromatin present outside the nucleus that are formed via a nuclear-cytoplasmic blebbing process (Ivanov et al., 2013). CCFs induce an innate immune response by activating the cytosolic DNA sensing cyclic GMP–AMP synthase (cGAS)-STING pathway (Dou et al., 2017; Gluck et al., 2017). This in turn promotes the SASP via activation of the NF-κB pathway, leading to a pro-inflammatory response (Vizioli et al., 2020; Miller et al., 2021).

### Secretory Phenotype

Senescent cells characteristically affect their surrounding tissue by secreting substances, grouped under the term senescence-associated secretory phenotype (SASP). This secretion is regulated by pathways involving the protein kinase p38MAPK and the transcription factor nuclear factor kappa beta (NF-κB). The secreted substances include soluble, growth and extracellular matrix (ECM)-remodeling factors, although recently other components such as extracellular vesicles and metabolites have been found (Fafian-Labora and O'Loghlen, 2020). They act by either reinforcing or spreading senescence to surrounding cells and activating immune responses for cell clearance. Soluble factors include interleukins (IL) 1α, 1β, 6, 7, 8, 13 and 15, and chemokines, such as monocyte chemoattractant proteins (MCP) 2 and 4, macrophage inflammatory proteins (MIP) 1a and 3a, and eotaxin. Such factors recruit immune cells to clear senescent cells. Growth factors include, among others, insulin-like growth factor (IGF), epidermal growth factor (EGF), fibroblast growth factor (FGF), vascular endothelial growth factor (VEGF) and angionenin. These factors signal pathways regulating cell growth and angiogenesis, and in such microenvironments, they contribute to senescence (Coppe et al., 2010). ECM-remodeling factors, such as matrix metalloproteinases (MMP), ADAMTS proteins and integrins also contribute to senescence. They act by altering ECM components, like collagen, and promote ECM degradation. This disrupts the normal crosstalk between cells and disorganizes their physical attachments, contributing to senescence. ECM disruption also aids in immune cell recruitment (Rapisarda et al., 2017; Fane and Weeraratna, 2020; Levi et al., 2020).

Extracellular vesicles are lipid membrane vesicles released by either pinching of the cytoplasmic membrane of the cell or formed upon activation of the endocytic pathway (van Niel et al., 2018). They contain a variety of proteins, nucleic acids, metabolites and lipids that decide their course of action and functionality in recipient cells (Tkach and Thery, 2016; O'Loghlen, 2018; O'Loghlen, 2022). They have been recently identified as part of the SASP mediating both paracrine senescence in different contexts (Borghesan et al., 2019; Jeon et al., 2019) and rejuvenation of different tissues in aged mice (Yoshida et al., 2019; Fafian-Labora et al., 2020; O'Loghlen, 2022).

Metabolites are an emerging part of the SASP and are discussed in the next section (Fafian-Labora and O'Loghlen, 2020; Smith et al., 2020).

### Deregulated Metabolism

Normal metabolism of the cell is also affected in the state of senescence leading to altered metabolite phenotypes (Smith et al., 2020). For example, the biosynthesis of leukotrienes is enhanced during senescence leading to their enrichment in the SASP which in turn promote lung fibrosis (Wiley et al., 2019). Importantly, it has been recently described that prostaglandin D2 is a useful biomarker to determine the efficacy of drugs that selectively eliminate senescent cells (termed senolytics) (Wiley and Campisi, 2021; Wiley et al., 2021).

As mentioned, malfunctioning mitochondria and anomalies in oxidative phosphorylation (OXPHOS) are characteristic of senescence (Dorr et al., 2013). Specifically, there is an increase in mitochondrial membrane potential during senescence that correlates with an increase in ROS production. This was partially described to be due to an increase in the expression of fatty acid synthase (FASN) (Fafian-Labora et al., 2019). Furthermore, senescence can cause mitochondria to alter their normal morphology. Examples include an increase in mitochondrial size and mass, mitochondria fusing together and fragments of mitochondria accumulating (Helman et al., 2016; Borghesan et al., 2019). In fact, mitochondrial dysfunction-associated senescence (MiDAS) is mainly driven by accumulation of NADPH in the cytoplasm, lowering NAD^+^/NADPH ratio, ATP levels and activating AMPK (Wiley et al., 2016). Furthermore, mitochondria elimination by mitophagy prevents both the cell cycle arrest and release of the SASP (Correia-Melo et al., 2016). Interestingly, functional mitochondria can be trafficked via extracellular vesicles, adding a layer of complexity to their role in cellular biology (Peruzzotti-Jametti et al., 2021).

Cellular granularity is a term used for any dense substance accumulating in cells and is a result of deregulated metabolism. Specifically, in senescence, lysosomal parts, protein aggregates such as amyloid-beta and proteolysed histones, and vesicles used for exocytosis are the main components of this granularity. In specific cell-types, like liver and muscle cells, glycogen granules are also highly abundant during senescence (Kwon et al., 2019).

## Senescence in Pathology

**FIGURE 2:**
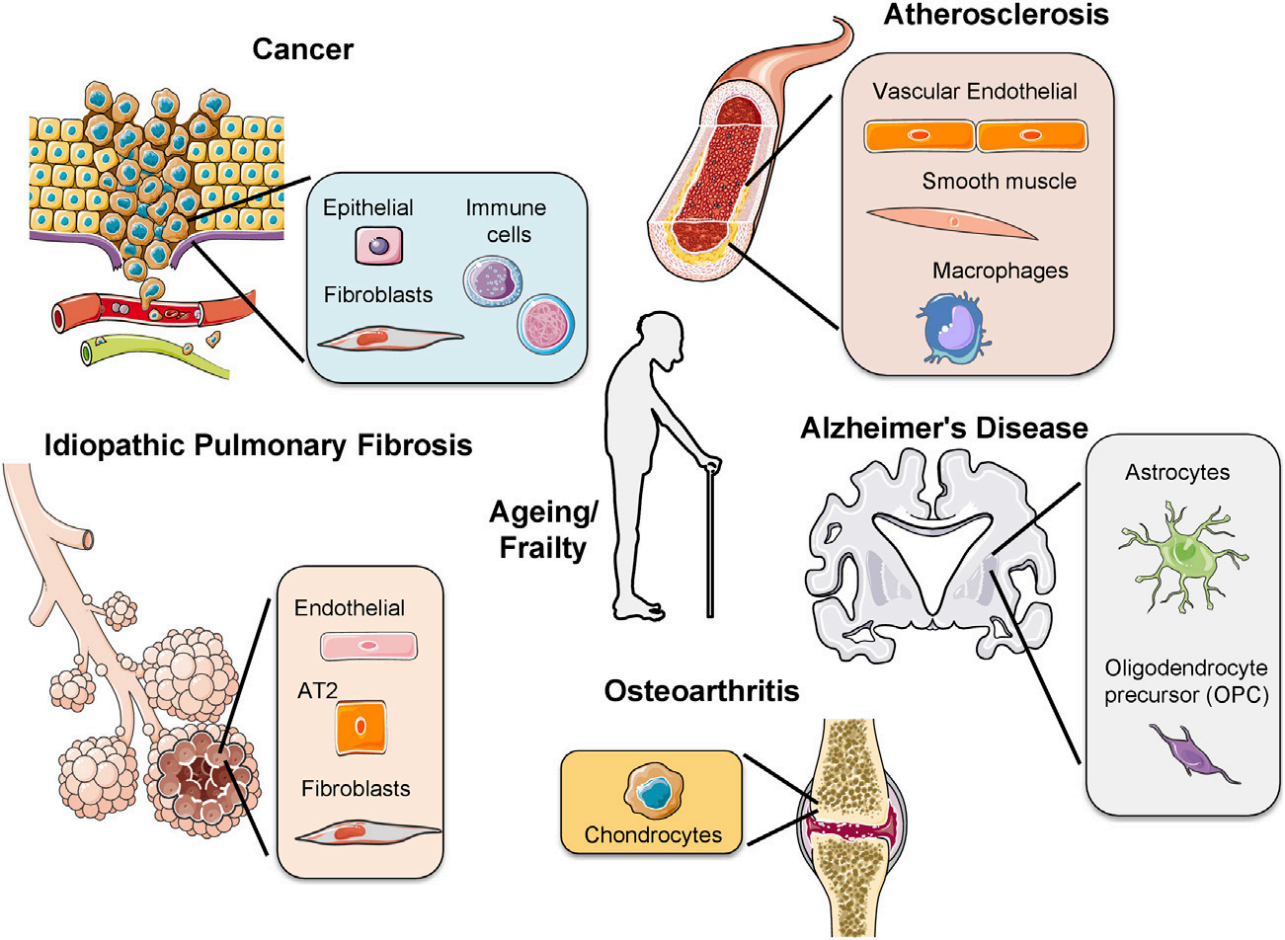
**Activation of senescence in different age-related pathologies**. The presence of senescent cells has been observed in different diseases, for example, osteoarthritis, idiopathic pulmonary fibrosis, cancer, atherosclerosis, Alzheimer’s disease and during frailty. In each disease different cell types undergo senescence. In cancer several immune cells from different origins as lymphoid or myeloid undergo senescence. AT2: alveolar type 2 cells.

Cells from each cell-type have a specific number of divisions, a principle called the Hayflick Limit (Hayflick and Moorhead, 1961). Stem cells have a larger cell-division capacity than differentiated cells. This is because they contain an enzyme, called telomerase, reversing telomere loss in each replication. Through human life-time, stem cells differentiate into tissue-specific cells to replace damaged cells. However, when stem cells reach their limit, they also undergo senescence and the tissue starts to age. Apart from ageing, early-onset of senescence due to oncogene activation or increased ROS formation causes disease (Munoz-Espin and Serrano, 2014). Here, we will discuss the implications of cellular senescence during ageing and in age-related pathologies (Figure 2).

### Ageing

Senescent cells increase in numbers as the organism ages. Such accumulation of senescent cells may impact on ageing through two possible mechanisms. First, the accumulation of senescent cells in tissues may disrupt tissue functionality, and, second, senescence may limit the regenerative potential of adult stem cells by disrupting the normal regulation of the stem cell niche within the tissue (van Deursen, 2014).

As mentioned previously, senescent cells secrete factors acting as paracrine signals to nearby cells and spreading senescence. These paracrine signals activate the recruitment of immune cells creating an inflammatory microenvironment (Fane and Weeraratna, 2020; Di Micco et al., 2021). Senescent signals not only damage proliferative cells but also stem cells inducing their rapid and abnormal differentiation into committed cells with dysfunctional characteristics (Paul et al., 2021). Additionally, during tissue repair, some parts of the tissue are replaced with fibrous materials in a process called tissue fibrosis in which the activation of senescence is crucial (van Deursen, 2014).

Diseases of the ageing population become more common, as human lifespan increases. Osteoarthritis, idiopathic pulmonary fibrosis, atherosclerosis and Alzheimer’s disease are, among others, characteristic examples of age-related diseases where senescence activation has been observed (McHugh and Gil, 2018).

### Osteoarthritis

Osteoarthritis is an age-related disease of cartilage loss in joints. Chondrocytes, the main cells in cartilage, are shown to undergo senescence. This is evident as osteoarthritic chondrocytes express p16^INK4A^ and the surrounding tissue within the affected joint has high concentrations of IL-1, IL-6 and MMP-3. In current research, procedures aiming to remove senescent cells from the tissue are being tested as a treatment option (Jeon et al., 2017; Coryell et al., 2021). Such processes are grouped under the term “senolysis” (Kelly, 2017; Dolgin, 2020). Furthermore, there is a group of drugs called “senomorphics” that act by targeting SASP factors, neutralising or inhibiting their actions (Gasek et al., 2021). For example, in mouse models for osteoarthritis, UBX0101 was used as an inhibitor for the MDM2/p53 interaction. The drug was able to eliminate the mice’s senescent chondrocytes providing some evidence for senomorphics as a future therapeutic option for osteoarthritis (Zhang et al., 2022).

### Idiopathic Pulmonary Fibrosis

Idiopathic pulmonary fibrosis (IPF) is a lung disease, correlated with advanced age in incidence and severity. The median survival is 2–3 years. Three different cell types have shown positive senescent markers in IPF: alveolar type 2 cells (AT2Cs), endothelial cells and fibroblasts. Such markers include p21^CIP1^, p16^INK4A^ and SA-β-Gal. AT2Cs are progenitor cells capable of self-renewal and differentiation into alveolar type 1 cells. Thus, they cause structural alterations in tissue architecture with each regeneration, contributing to disease. Fibroblasts are also important cells in the lungs, responsible for producing and maintaining the ECM. Thus, senescent fibroblasts remodel ECM and create a senescent microenvironment, furtherly progressing the severity of the disease (Schneider et al., 2021). Senomorphics have also been studied *in vitro* to show whether they can act as a therapeutic option for IPF. Specifically, the macrolide antibiotic roxithromycin managed to block the TGFβ stimulation, an important component of SASP, preventing further senescence induction to lung fibroblasts (Zhang et al., 2021).

### Atherosclerosis

Atherosclerosis is a disease of human arteries, where a fatty streak is formed and occludes the vessel. The development of atherosclerosis is related to the presence of oxidised low-density lipoprotein (LDL) in the vessel wall, chronic inflammation and malfunctioning vascular endothelial cells. Vascular endothelial cells in atherosclerotic coronary arteries have shown positive SA-β-Gal activity and expression of p21^CIP1^ and p16^INK4A^, suggesting that they undergo senescence. Senescence creates a leaky and highly permeable endothelium, allowing macrophages and other immune cells to enter. Macrophages have a crucial role in atherosclerosis. They enter the ECM of the vessel wall, induce ECM degradation and absorb the oxidised LDL, becoming part of the fatty streak. Smooth muscle cells in vessels also undergo senescence, enhancing the hazardous microenvironment by their SASP and forming the necrotic core of the fatty streak. Finally, the recruitment of monocytes and macrophages by SASP, and the high abundance of senescent macrophages in the organism are both contributing to atherosclerosis progression (Minamino et al., 2002; Bennett and Clarke, 2016; Bennett and Clarke, 2017). Drugs with senomorphic properties have also been studied for their effectiveness into treating atherosclerosis. For example, drugs like rapamycin and ruxolitinib inhibit intracellular mediators, such as the kinase mTOR, that play a key role in SASP initiation (Ramírez et al., 2022).

### Alzheimer’s Disease

Alzheimer’s disease is characterised by extracellular deposition of amyloid-beta (Aβ) plaques. As mentioned above, amyloid-beta is part of the protein aggregates that form in senescent cells. Astrocytes, cells responsible for regulating the homeostasis of the neurons and maintaining the blood-brain barrier, are involved in the pathology of the disease. Astrocytes expressing p21^CIP1^, p16^INK4A^ and active SA-β-GAL are related to an increased Aβ plaque formation. Specifically, senescent astrocytes and their SASP limit the clearance of Aβ deposits by microglia. Also, exogenous application of Aβ oligomers can trigger astrocytes to undergo senescence. Additionally, SASP from astrocytes can induce neurofibrillary tangle formation in neurons, another common feature of Alzheimer’s disease. In addition, the presence of senescence markers has also been observed in oligodendrocyte progenitor cells (OPCs) where treatment with senolytics alleviates cognitive deficits in an Alzheimer’s disease model (Zhang et al., 2019). Altogether, these results suggest there is a connection between senescence and Alzheimer’s disease pathogenesis (Saez-Atienzar and Masliah, 2020; Guerrero et al., 2021). Senomorphic agents against Alzheimer’s disease have been studied even in human trials. For example, dasatinib and quercetin have been shown to interact with AKT, an important protein involved in the regulation of the SASP (Zhang et al., 2022).

### Frailty

Frailty is a clinical state characterised by a low overall physical activity, a decline in multiple physiological systems and an increased vulnerability to stress-inducing factors mostly resulting from physiological ageing. It is associated with -but not defined by- increased chronological age and multiple comorbidities. The frail phenotype presents mainly with a noticeable decrease in muscle strength, general weakness, a significant decrease in walking speed, unintentional weight loss and reports of exhaustion (Fried et al., 2001).

Senescence is associated with the pathophysiology of many chronic conditions that are considered as variables for assessing a Frailty Index, such as Alzheimer’s disease. Furthermore, the frail phenotype can also be considered as a result of cellular senescence (LeBrasseur et al., 2015). Systemic inflammation is one of the main reasons that frail individuals feel general weakness and exhaustion. Accumulation of senescent cells is known to dysregulate the response of the immune cells present in the tissue’s microenvironment and cause significant changes to the secretions of inflammatory factors. For example, IL-6 and TNF-α secretions by senescent and immune cells are increased in aged tissues and their receptors are upregulated on various cell types. When this pathophysiological response is repeated several times and in multiple tissues, the individual reaches a long-term unresolved chronic inflammatory state which acts as a precipitant of frailty (Sathyan et al., 2020). Additionally, the decline in strength can also be attributed to senescent cells disrupting the normal neuromuscular physiology. Muscle mass loss (sarcopenia) and denervation or neuron loss within neuromuscular junctions are mostly due to increased senescence induction and insufficient clearance of senescent cells (Sousa-Victor et al., 2021). Finally, senescence resulting in inflammation and deterioration of brain function causes mental decline contributing to the inability of a frail individual to perform everyday tasks (Guerrero et al., 2021). Senescence induction in the neurons, the brain’s immune cells and the vascular cells of the brain’s blood vessels result in the development of neurologic and psychological conditions causing disorientation, difficulty with planning, mood and personality changes, emotional decline and many other symptoms that decrease the person’s ability to perform simple tasks.

In conclusion, it is important to make clear that senescence is what many use to define physiological ageing, the biggest contributor to frailty. Physiological age differs from chronological age, which is simply the number of years a person is alive. Physiological age is a result of environmental, genetic and epigenetic factors that lead to the ageing of tissues and the deterioration of their functions, which progressively causes the individual to reach the state of frailty.

### Cancer

Cancer is a disease affecting 17 million people every year and is responsible for 9.6 million deaths. Cancer arises from a single cell that undergoes several mutations in genes regulating cell proliferation and division. This cell then grows and divides indefinitely, forming a three-dimensional structure, the tumour. Cancerous cells compete with healthy cells for resources and they succeed due to their advantageous mutations favouring cell cycle progression (Hanahan and Weinberg, 2011; Leite de Oliveira and Bernards, 2018).

Senescence has a crucial role in preventing cancer development. Cancer cells must proliferate rapidly and indefinitely. Thus, a stable cycle-arrest causes restriction of tumour growth and prevention of further cell division. The SASP also contributes to anti-tumour activity. It facilitates the recruitment of immune cells through the expression of several cytokines and interleukins, promoting a systemic response to eradicate cancer (Leite de Oliveira and Bernards, 2018; Lee and Schmitt, 2019). Furthermore, even cells of progressed cancer can enter the senescent state (Lee and Schmitt, 2019). This can be induced by several rounds of chemotherapy treatment (Leite de Oliveira and Bernards, 2018). Although paradoxically, senescence also has shown to have pro-tumorigenic activity, especially in promoting cancer relapse following chemotherapy (Demaria et al., 2017).

Several mechanisms are used by cancerous cells to evade senescence activation and disrupt cell cycle arrest, promoting cell cycle progression. Inactivation of the p27^KIP1^ is an important step of such mechanisms. There are mutations that favor the activation of proteins promoting the function of the CyclinE-CDK2 complex, phosphorylating p27^KIP1^ and tagging it for degradation. This represses cyclin D, allowing cell cycle progression, favouring cancer cell proliferation (Hanahan and Weinberg, 2011). On the other hand, AKT, a serine/threonine kinase, can also inhibit p27^KIP1^ and promote cell cycle progression. The activity of AKT can be upregulated directly by mutations causing overexpression of the AKT gene. AKT signalling can also be upregulated indirectly by mutations causing a decrease in the exogenous TGFβ stimulation or an increase in stimuli from exogenous mitogens. These changes in exogenous stimuli activate phosphatidylinositol 3-kinase (PI3K), which activates AKT. AKT then acts by translocating p21^CIP1^ and p27 ^KIP1^ from the nucleus to the cytoplasm, where they are unable to inhibit the proteins that promote cell cycle progression (Hanahan and Weinberg, 2011).

A key characteristic of cancer cells is the deregulation of cell cycle checkpoint proteins such as the cyclin-dependent kinases (CDKs) CDK4 and CDK6 leading to uncontrolled cell proliferation. Molecular changes at CDKs level have been reported in various cancer types making them an attractive potential target for new treatments. CDK inhibitors, in particular CDK4/6 inhibitors (Abemaciclib, Palbociclib and Ribociclib), induce cell-cycle arrest and subsequent senescence in several cancer cell lines and mouse models (Asghar et al., 2015; Alvarez-Fernandez and Malumbres, 2020; Carpintero-Fernandez et al., 2022). Palbociclib is a drug for oestrogen-receptor-positive breast cancer (ER^+^). ER^+^ is the type of breast cancer where cancerous cells proliferate in response to oestrogen stimulation. Palbociclib acts by selectively inhibiting CDK4/6, leading to the accumulation of unphosphorylated Rb finally inducing cell cycle arrest (Clark et al., 2016). Palbociclib combined with letrozole or other aromatase inhibitors was approved for breast cancer treatment in 2015. Since then, Palbociclib has shown positive therapeutic outcomes in gastric cancer, melanoma, liposarcoma and hepatocellular carcinoma (Finn et al., 2015).

The fact that evasion of senescence is an important step for cancer development lead to the hypothesis that re-activating senescence in cancerous cells can reduce tumour progression. Thus, the discovery of drugs that can induce senescence in cancer cells can be a major contribution to the research field of cancer treatment. On the other hand, a great effort is currently ongoing to develop drugs that selectively eliminate senescent cells -senolytics. It has been shown that the use of senolytics in cancer prevents cancer progression in a variety of mouse models (Dolgin, 2020). Importantly, the group of Scott Lowe has recently developed a more specific technique to selectively eliminate senescent cells: via the generation of a Chimeric Antigen Receptor (CAR construct) targeting the urokinase-type plasminogen activator receptor (uPAR) which is expressed in senescent cells. Using this technology the authors where able to ablate senescent cells in mouse models of lung cancer and liver fibrosis (Amor et al., 2020).

## Controversies and Unknowns In The Senescence Field

In spite of the huge scientific advances made in the last decades since the discovery of senescence in the early 1960s, there are still many open questions in the field. One example is the heterogeneity present upon the induction of senescence, where different cells are at different states in the activation of this phenotype. This added to the fact that a variety of specific but uncharacterized biomarkers can be induced makes it extremely difficult not only for their identification but also for their pharmacological targeting via senolytics and senomorphics (Hernandez-Segura et al., 2017). Additionally, evidence is building up that senescence is a stable, but not irreversible, cellular state. In fact, this novel characteristic of senescent cells has been attributed to the plasticity of their SASP in different contexts such as cancer, reprogramming and rejuvenation (Mosteiro et al., 2016; Ocampo et al., 2016; Ritschka et al., 2017; Rhinn et al., 2019; Fafian-Labora et al., 2020; Carpintero-Fernandez et al 2022). Importantly, recent evidences show that the SASP is not unique and static as initially thought but that it is dynamic, changing with time depending on trigger, context and cellular type (Hoare et al., 2016; Lee and Schmitt, 2019). The functions these variable SASP components have on their surroundings and specially on their interaction with the immune system is currently under thorough scrutiny.

## Conclusion

In summary, the presence of senescent cells has been identified in numerous diseases related to ageing. In addition, their partial contribution to deleterious effects on the organism is starting to be well documented. Altogether, this brings light into the perspective that ageing and age-related diseases can be cured or at least delayed by human interventions. However, the fact that different cell types undergo senescence, that there is no single gold standard marker to identify senescence and that the markers of senescent cells most likely change depending on the microenvironment and with time makes therapeutic treatment very challenging. This in addition to the fact that the activation of senescence is beneficial in certain circumstances and for several pathologies. Thus, more research effort is required to deeply understand this complex cellular phenotype and how it influences the microenvironment, tissue and organism.
